# Profiling of rumen fermentation, microbial population and digestibility in goats fed with dietary oils containing different fatty acids

**DOI:** 10.1186/s12917-018-1672-0

**Published:** 2018-11-14

**Authors:** I. Nur Atikah, A. R. Alimon, H. Yaakub, N. Abdullah, M. F. Jahromi, M. Ivan, A. A. Samsudin

**Affiliations:** 1 Institute of Tropical Agriculture, Universiti Putra Malaysia, UPM, 43400 Serdang, Selangor Malaysia; 2 Department of Animal Science, Faculty of Agriculture, Universiti Putra Malaysia, UPM, 43400 Serdang, Selangor Malaysia

**Keywords:** Dietary oil, Digestibility, Goat, Rumen fermentation, Rumen microbial population

## Abstract

**Background:**

The effects of the dietary oils with differing fatty acid profiles on rumen fermentation, microbial population, and digestibility in goats were investigated. In Experiment I, rumen microbial population and fermentation profiles were evaluated on 16 fistulated male goats that were randomly assigned to four treatment groups: i) control (CNT), ii) olive oil (OL), iii) palm olein oil (PO), and iv) sunflower oil (SF). In Experiment II, another group of 16 male goats was randomly assigned to the same dietary treatments for digestibility determination.

**Results:**

Rumen ammonia concentration was higher in CNT group compared to treatment groups receiving dietary oils. The total VFA and acetate concentration were higher in SF and OL groups, which showed that they were significantly affected by the dietary treatments. There were no differences in total microbial population. However, fibre degrading bacteria populations were affected by the interaction between treatment and day of sampling. Significant differences were observed in apparent digestibility of crude protein and ether extract of treatment groups containing dietary oils compared to the control group.

**Conclusions:**

This study demonstrated that supplementation of different dietary oils containing different fatty acid profiles improved rumen fermentation by reducing ammonia concentration and increasing total VFA concentration, altering fibre degrading bacteria population, and improving apparent digestibility of crude protein and ether extract.

## Background

Ruminant acquires energy from plant materials through the activity of microbial fermentation and plant degradation mainly by groups of cellulolytic bacteria. The dynamics of major cellulolytic bacterial population found in the rumen, in particular *Fibrobacter succinogens, Ruminococcus albus,* and *Ruminococcus flavefaciens,* have been widely studied in response to dietary shift [1] or between species [2] using molecular approaches of quantitative real-time polymerase chain reaction (qPCR). The importance of cellulolytic bacteria in ruminant nutrition is due to the fact that this particular group of bacteria plays a critical role not only in utilizing feeds that are not suitable for monogastric animals, but also in facilitating animals to survive on poor quality fibrous forages [3]. During the fermentation process, energy is released in the form of adenosine triphosphate (ATP), which is used to fuel different activities of rumen microorganism. This energy can be improved in ruminant by supplementing the animals with dietary fat, an approach that has been commonly practiced.

Other studies have shown different effects of vegetable oil supplementations in rumen fermentation and microbial population using cattle. For example, [4] reported that supplementation of linseed oil to dairy cow did not affect ruminal pH, ammonia, and total volatile FA concentrations. Similarly, [5] reported that flaxseed supplementation to calves has no effects on rumen fermentation parameters. However, fish oil supplementation in steer ruminal fluid had lower ruminal acetate and butyrate but greater propionate concentration, as reported by [6]. Another study from [7] reported that feeding oilseeds from flaxseed to cows had no effects on pH and concentrations of NH_3_-N and total volatile fatty acids while acetate: propionate ratio was decreased. In the same study, they also described that oilseeds decreased protozoa and increased total cellulolytic bacteria population in rumen fluid. Similarly, [8] showed that supplementation with palm oil in cows reduced protozoa population, but it did not affect cellulolytic bacteria population in cows. Other vegetable oils such as coconut oil [9] and soybean oil [10] are used as energy sources and have the potential to manipulate the microbial ecosystem of the rumen to enhance fibrous feed digestibility, reduce methane emission, and reduce nitrogen excretion by ruminants [11].

There is not much information available that emphasizes the effect of diets supplemented with olive oil that contains oleic acid (C18:1), sunflower oil that contains linoleic acid (C18:2), and palm olein oil that consists of linoleic acid (C18:2) and palmitic acid (C16:0) on rumen fermentation, rumen microbial populations, and digestibility in goats. Therefore, the aim of this study was to investigate rumen microbial population, fermentation profile and nutrient digestibility for local goats fed diets supplemented with sunflower oil (SF), olive oil (OL), or palm olein oil (PO).

## Results

### Rumen pH and volatile fatty acids

The results of ruminal pH and VFA concentration are presented in Table 1. The mean of ruminal pH ranged between 6.26 (PO) and 6.80 (OL) and was affected by day of sampling (*P* < 0.01). Different treatment diets had no significant effect on ruminal pH. However, OL fed group tended to have a slightly higher rumen pH value than that of the other groups.

**Table 1:** Rumen fermentation parameters (mean ± SE) of goats fed diet supplemented with different types of oils

Parameters	Treatment				*P*-value		
	CNT	OL	PO	SF	Tr	Day	Tr × Day
pH	6.29 ± 0.11	6.80 ± 0.11	6.26 ± 0.06	6.33 ± 0.07	NS	**	NS
Ammonia (mg/l)	42.6 ± 1.65^a^	37.9 ± 1.36^b^	36.4 ± 1.08^b^	36.9 ± 1.24^b^	**	*	*
Total VFA (mmol)	89.42 ± 8.59^b^	95.58 ± 7.81^a^	91.11 ± 6.44^b^	95.79 ± 3.15^a^	*	*	NS
Acetate (%)	59.22 ± 5.83 ^b^	63.70 ± 5.46^a^	56.79 3.56^b^	63.72 ± 2.01^a^	*	*	*
Propionate (%)	18.21 ± 2.11	19.62 ± 1.47	22.15 ± 2.81	20.22 ± 0.95	NS	NS	NS
Butyrate (%)	8.19 ± 0.89	8.83 ± 0.99	8.13 ± 0.96	8.37 ± 0.71	NS	*	NS
Isobutyrate (%)	0.76 ± 0.13^b^	0.88 ± 0.11 ^a^	0.73 ± 0.04^b^	0.68 ± 0.04^b^	*	NS	NS
Valerate (%)	2.18 ± 0.48	2.42 ± 0.13	2.14 ± 0.24	1.72 ± 0.28	NS	*	*
Isovalerate (%)	0.86 ± 0.25	1.13 ± 0.13	1.17 ± 0.14	1.08 ± 0.16	NS	NS	NS
Acetate/Propionate	3.25 ± 0.18	3.72 ± 0.22	2.56 ± 0.30	3.15 ± 0.19	NS	NS	NS

*CNT* Control diet, *OL* Olive oil diet, *PO* Palm olein diet, *SF* Sunflower oil diet, *Tr* Treatment

^*^Significant level at *P* < 0.05; ^**^ Significant level at *P* < 0.01

^a, b^Means in the same row with different superscripts are statistically different (P < 0.05)

The total VFA concentration (mmol) was significantly higher (*P* < 0.05) in OL and SF compared to the CNT and PO groups (Table 1). The OL and SF groups had higher (*P* < 0.05) concentration of acetic acid compared to the other groups. Diets supplemented with PO did not show significant differences in acetate when compared to the control group. Supplementation of OL showed an increased level of isobutyric acid concentration when compared to other treatment groups. Propionate, butyrate, valerate, and isovalerate concentrations and acetic acid to propionic acid ratio (A/P) were not affected by the different types of oil supplementation. However, the significant effect of sampling day can be observed in the concentrations of the total VFA, acetate, butyrate, and valerate.

### Ammonia

The mean values of ruminal ammonia are presented in Table 1. Addition of dietary oils significantly decreased the concentration of ruminal ammonia. The concentration (mg/l) of ammonia-N in the rumen fluid was significantly affected by diet (*P* < 0.05), day of sampling (*P* < 0.01), and diet × day of sampling interaction (*P* < 0.05). Higher amount of ammonia concentration was observed in CNT (42.6 mg/l) compared to other groups (36.4–37.9 mg/l).

### Microbial population

The effects of dietary oils on rumen microbial population are presented in Table 2. Higher numbers of total bacteria could be observed in the treatment groups compared to CNT although not statistically significant (*P* > 0.05). No significant difference was observed in the *F. succinogenes, R. albus,* and *R. flavefaciens* populations although the highest level of log10 copy no./g was recorded in SF groups. Significant differences (*P* < 0.05) were observed for methanogenic archea population, and the numbers were influenced by treatment, day of sampling, and the interaction of treatment × day. Population of protozoa demonstrated that there were no significant differences among all the treatment groups although the CNT and PO groups had higher values of log10 copy no./g and were significantly affected by day of sampling (*P* < 0.05). As shown in Table 2, significant differences are seen in the methanogens population while the protozoa population is not significantly affected by the treatment diets.

**Table 2:** Effects of supplementation with different types of oils on microbial population (mean ± SE) in the rumen of goats

Parameter	Treatment				*P*-value		
	CNT	OL	PO	SF	Treatment	Day	Treatment×Day
Total Microbes (Log10 copy No/g)	9.56 ± 0.21	10.20 ± 0.15	10.01 ± 0.13	9.83 ± 0.16	NS	NS	NS
*Fibrobacter succinogenes* (Log10 copy No/g)	4.20 ± 0.18	3.67 ± 0.18	4.20 ± 0.14	4.21 ± 0.14	NS	*	*
*Rumonococcus albus* (Log10 copy No/g)	7.81 ± 0.21	7.65 ± 0.18	7.95 ± 0.22	8.07 ± 0.19	NS	*	*
*Ruminococcus flavefaciens* (Log10 copy No/g)	5.02 ± 0.19	5.06 ± 0.26	5.32 ± 0.20	5.36 ± 0.10	NS	*	*
Methanogenic archea (Log10 copy No/g)	3.60 ± 0.12^b^	4.23 ± 0.22^a^	3.91 ± 0.14^ab^	4.39 ± 0.21^a^	*	*	*
Protozoa (Log10 copy No/g)	3.30 ± 0.22	2.63 ± 0.22	3.30 ± 0.13	3.22 ± 0.15	NS	*	NS

*CNT* Control diet, *OL* Olive oil diet, *PO* Palm olein diet, *SF* Sunflower oil diet, *Tr* Treatment

^*^Significant level at *P* < 0.05

^a, b^Means ± std. error in the same row with different superscripts are statistically different (*P* < 0.05)

### Apparent digestibility

The results of apparent digestibility study are presented in Table 3. The DM, OM, NDF, and ADF also followed a similar pattern with the treatment group (*P* > 0.05). The CP apparent digestibility was significantly improved (*P* < 0.05) in all treatment groups containing oil, in which OL had the highest CP apparent digestibility (85.04%). The apparent digestibility of EE also followed a similar pattern where the three treatments of OL, PO, and SF had a higher EE digestibility (*P* < 0.05) compared to CNT, with SF groups having the highest apparent digestibility percentage (91.13%). However, no significant difference was observed in fibre digestibility although the PO and SF groups numerically tended to have higher ADF digestibility.

**Table 3:** Apparent digestibility (% DM) of nutrient (mean ± SE) in goats fed diet supplemented with different types of oils

Apparent digestibility (%)	Treatment				*P*-value
	CNT	OL	PO	SF	
Dry matter	75.81 ± 2.77	73.49 ± 1.98	75.44 ± 1.09	75.37 ± 1.57	NS
Organic matter	80.05 ± 1.78	80.84 ± 1.57	80.17 ± 1.61	78.81 ± 2.34	NS
Crude protein	77.65 ± 0.81^b^	85.04 ± 1.19^a^	82.20 ± 2.27^a^	82.43 ± 1.44^a^	*
Ether extract	59.89 ± 10.39^b^	88.11 ± 1.46^a^	87.92 ± 1.93^a^	91.13 ± 2.76^a^	*
NDF	75.19 ± 4.31	71.59 ± 2.84	71.82 ± 3.47	71.28 ± 4.31	NS
ADF	47.42 ± 1.99^b^	54.75 ± 5.27^a^	57.54 ± 4.49^a^	55.01 ± 4.41^a^	*

*CNT* Control diet, *OL* Olive oil diet, *PO* Palm olein diet, *SF* Sunflower oil diet

^a, b^Means in the same row with different superscripts are statistically different (*P* < 0.05)

## Discussion

### Rumen pH and volatile fatty acids

Ruminal pH values were within normal range, and the increment has minimal effects on rumen cellulolytic processes of fibre and protein digestion (6.0–7.0) [12]. This result suggests that the microbial population of rumen is able to adapt to the diet given, regardless of the additions and differences in composition of dietary oil supplemented [13]. Adequate roughage supply in the diet reduced the negative effect of dietary oil on rumen fermentation because the fibre fraction creates a supporting environment for rumen microbes to hydrolyze the dietary oils [6, 14, 15]. The findings of the present study are consistent with those of [8] who reported that supplementation of palm oil did not give negative effects on ruminal pH in dairy cows. In addition, other studies using different types of dietary oils in other ruminants have also reported similar observations [16–18].

The increased level of the total VFA concentration in OL and SF groups in the present study indicates the efficiency of nutrient digestion. It confirms the fact that the notable effect of supplementing dietary oils on rumen fermentation depends on the type and level of fatty acids [10]. A similar result was reported by [19], where supplementation of C_18_ fatty acid increased the total VFA concentration although less influence was seen in the different types of fatty acid supplemented. Nevertheless, different responses were observed in the work done by [20] that showed the reduction in the concentration of VFA supplemented with fatty acids.

A significant increase (*P* < 0.05) in the acetate level in the OL and SF can also suggest a modification of the ruminal microbial population [19, 21]. However, the validation on this reason needs to be done in future studies. This result is thought to be due to the modification of the ruminal microbial ecosystem, as occurs with 18-carbon polyunsaturated FA. A decrease in cellulolytic and methanogenic bacteria is observed with most fat sources inclusion. For branched fatty acid concentration, there was a significant increment in the molar proportion of isobutyrate in the OL group. The finding is consistent with a study done by [20] who reported that branched fatty acid concentration was increased with the supplementation of fatty acid.

### Ammonia

Fat supplementation in ruminant diets has been shown to consistently depress rumen ammonia concentration [22, 23]. In the current study, the ammonia level recorded in the oil supplemented groups was within the normal range as reported by [24]. The optimum ammonia level that favors the ruminal microbial activity in animals fed with lignocellulosic materials was between 16.5 and 37.9 mg/l. There were also significant differences observed by the interaction of treatments with day of sampling, suggesting that ammonia level in rumen might be associated with the shift of the microbial population of rumen by time, due to the addition of dietary oils. A report by [25] suggested that the increase in the ammonia level was due to the reduction in protozoal predation toward rumen bacteria thus, reducing the recycling of bacteria protein in the rumen. A similar result by [26] also reported that ammonia concentration tended to increase when the longer chain of unsaturated fatty acid was present in the diet. [27] reported that rumen ammonia concentration reduction corresponded with lowered ammonia flow to the duodenum and was similar to other studies [28, 29] in sheep as well as in cattle [18, 22]. However, contradictory results with regard to ammonia level in previous studies on ruminant have been noted with supplementations of linoleic acid by [19, 30] and supplementations of sunflower oil in cattle by [31].

### Microbial population

Supplementation of vegetables oils did not alter the fibre degrading bacteria and total microbial populations in the present study, indicating that these microorganisms are not sensitive to dietary oils supplementation [32]. Another possible reason was mentioned by [33] who reported that the negative effects toward ruminal fibrolytic bacteria were neglected in the case of high grain-fed diet. In their study, neither NDF nor fibrolytic bacteria population has shown significant responses toward fatty acid supplementation. Vegetable oil supplementations have shown inconsistent results of rumen microbial population in other studies. [6] observed a decrease of *F. succinogenes* population but not the *R. albus,* and *R. flavefaciens* populations in steers. Furthermore, [10] reported a decrease of *F. succinogenes* and *R. flavefaciens* population. In addition, [34] observed an increase of *F. succinogenes*, *R. albus,* and *R. flavefaciens* populations in goats. [32] on the other hand, observed no effects on *F. succinogenes* population and a decrease of *R. albus* and *R. flavefaciens* populations in goats. The significant differences observed by sampling day and interaction of treatments with sampling day on fibre degrading bacteria, methanogens, and protozoa populations in the present study suggest that the ruminal microbial populations shifted by time due to the addition of dietary oils.

The population of protozoa in rumen often correlates to the population of methanogens. It has been reported that a reduction in protozoa reduced the methanogens population since methanogens live in association with protozoa, linked by hydrogen transfer within the interspecies [35]. However, the association of those methanogens is only 0.1–0.2% of the total population, whereby the others that exist freely in the rumen might not be affected by the supplementation to the same extent. Due to that, the reduction of methanogens does not always follow the population pattern similar to protozoa. A decrease in rumen protozoa population was observed with the supplementation of blended canola and palm oil by [34] and supplementation of linseed oil and coconut oil by [36]. A similar observation was also reported by [10] who observed that dietary soybean oil reduced the population of methanogens in lambs.

### Apparent digestibility

An increase in CP digestibility in the treatment groups suggests that oil supplementation can act as a source of energy for rumen microbes to convert feed protein into microbial protein, which is more digestible. Besides, the increased CP digestibility may be due to the reduction in the microbial degradation by protozoa, which in turn increased the level of protein available in the lower sections of the gastrointestinal tract [37].

The higher apparent digestibility of EE in the treatment groups reported in this study is in agreement with a study using lambs by [38]. Diet rich in dietary fats tends to have a higher hydrolysis percentage in the rumen compared to the conventional diet [39]. Lipases that are involved in rumen lipid hydrolysis have been shown to be more active in diets with high fibre and protein contents [40]. A previous study by [15] showed that fatty acid had higher digestibility with increasing number of double bonds. In this study, although there were no significant changes, the SF group showed higher values in EE digestibility. It may be due to the presence of linoleic acid (C18:2) in sunflower oil compared to the presence of oleic acid (C18:1) in both olive oil and palm olein.

Supplementation of dietary oils tended to coat the particle of fibre, thus preventing them from the attack of rumen microbes [41]. In the present study, although there was a decreased pattern of NDF digestibility, there were no significant differences observed. This may suggest that the level of oil inclusion is not enough to deliver the effect. This result is also supported by [42] who indicated that fibre digestion would be limited when fat content in ruminant diet is higher than 70 g/kg DM intake, a level which is higher than the level used in the present study. Another reason is that NDF digestibility follows ruminal protozoa population, as supported in a study by [43]. Similar results by [44] did not find variations in NDF digestibility whereas ADF digestibility was higher in in vitro study. The increase in nutrient digestibility observed in the present study might have been caused by the increase in ruminal retention time as suggested by [45, 46].

## Conclusion

Supplementation with olive oil, palm olein oil, and sunflower oil improved and developed better ruminal microorganism population to a certain extent in goats. In terms of digestibility, oil supplementation improved both protein and fat digestibility. For future research, it is recommended that studies on the effects of C_18_ fatty acids on the metabolic activity of microbial population in rumen, particularly methanogens, be deeply clarified, including determination of the long-term effects of fatty acids on in vivo rumen fermentation, methanogenesis, and animal performance. With regard to rumen physiology, understanding the association, symbiotic relationship, and cross feeding among microorganism is important in predicting the response of microorganisms when given a new diet.

## Methods

### Animals and diet

The experiment was carried out at Department of Animal Science, Faculty of Agriculture, Universiti Putra Malaysia following the guidelines approved by the Institutional Animal Care and Use Committee (IACUC) (Approval No. R064/2016) of the Universiti Putra Malaysia. Sixteen mature local Katjang-crossed male goats aged between 20 and 24 months with an average weight of 28.32 ± 1.85 kg and fitted with rumen cannula were used for rumen fermentation profile and microbial population study (Experiment I). The animals were properly maintained by treated against endo and ectoparasites prior to the commencement of the experimental procedure. In addition, all of the animals were under supervisory of veterinarian for assessing their health. In another study (Experiment II), 16 Katjang-crossed male goats aged between 10 and 12 months with an average weight of 23.17 ± 0.94 kg were used to determine the digestibility of nutrients in the diets. The diets were formulated to have approximately equal amount of crude protein (CP) and energy content [47]. All of the animals used in these two studies were purchased from a commercial farm, De Kebun Enterprise in Selangor.

The animals were randomly assigned into four groups to receive four different dietary treatments: i) basal diet (CNT or control), ii) basal diet + olive oil (OL), iii) basal diet + palm olein oil (PO), and iv) basal diet + sunflower oil (SF). The oil content was supplemented at the rate of 6% of the total feed ingredients. The oil content was supplemented at the rate of 6% of the total feed ingredients. The ingredients, including the oils, were purchased from commercial sources. Dry matter (DM), organic matter (OM), crude protein (CP), neutral detergent fibre (NDF), and acid detergent fibre (ADF) of the experimental diets were analyzed according to [48, 49]. The ingredients and chemical composition of the diets are presented in Table 4.

**Table 4:** Ingredients and chemical composition of treatment diets

	Treatments			
	CNT	OL	PO	SF
Ingredient (as fed)				
Rice straw	30.8	25.8	25.8	25.8
Barley grain	35.0	35.0	35.0	35.0
Soybean meal	30.0	30.0	30.0	30.0
Molases	2.0	1.0	1.0	1.0
Vitamin mineral-mix	0.5	0.5	0.5	0.5
Limestone	1.3	1.3	1.3	1.3
Sodium Sulphate	0.4	0.4	0.4	0.4
Olive oil	–	6.0	–	–
Palm Oil	–	–	6.0	–
Sunflower oil	–	–	–	6.0
Chemical analysis (DM %)				
DM	76.17	76.02	78.27	78.73
OM	93.60	93.34	94.61	94.96
CP	15.76	15.48	15.9	16.00
EE	1.86	4.56	4.70	4.74
NDF	63.53	58.76	58.54	51.27
ADF	17.04	18.26	20.66	21.41
Fatty acid (g/100 g total fatty acid DM)				
C-16:0	8.09	11.72	44.50	4.92
C-18:0	2.70	1.40	3.73	6.28
C-18:1, *n* 9	27.88	73.20	41.45	28.47
C-18:2, *n* 6	55.76	12.9	9.62	59.36
C-18:3, *n* 3	5.57	0.80	0.30	0.97

*CNT* Control diet, *OL* Olive oil diet, *PO* Palm olein diet, *SF* Sunflower oil diet, *DM* Dry matter, *OM* Organic matter, *CP* Crude protein, *EE* Ether extract, *NDF* Neutral detergent fiber, *ADF* Acid detergent fiber, *C-16:0* Palmitic acid, *C-18:0* Stearic acid, *C-18:1, n 9* Oleic acid, *C-18:2, n 6* Linoleic acid, *C-18:3, n 3* Linolenic acid

### Animal housing and management

The experiments were conducted in Serdang, Selangor, Malaysia (3° 2′ 0” North, 101° 43′ 0″ East). The cannulated animals were kept individually in separate pens while in the digestibility study, the animals were kept in an individual metabolic crate and had free access to water.

### Experimental procedure and sampling schedule

Experiment I was conducted for 28 days of adjustment period followed by 30 days of treatment period. During the adjustment period, all the animals were fed with basal diet that acted as the control diet. The diet was offered ad libitum to the goats at 09:00 daily. After the adjustment period, the animals were randomly assigned according to a completely randomized design into four groups, with each group consisted of four goats and received one of the four dietary treatments. The random selection was done in Microsoft Excel using tag number of animals. Rumen content was collected on days 27 and 28 of the adjustment period, which is considered as the initial sampling day. The data were pooled and recorded as day 0 of the experimental period. Rumen samples were collected from different parts of the rumen 2 h after morning feeding through the cannula. Similarly, rumen samples were also collected on day 2, 4, 6, 12, 18, 24, and 30 of treatment period.

Determination of rumen microbial population was done on the rumen content collected on day 0, 12, and 30 using real-time PCR. Briefly, about 500 ml of rumen fluid was collected. Rumen pH was immediately measured and then divided into two portions. The first portion of rumen fluid collected was squeezed through four layers of cheesecloth to eliminate larger solid feed particles. Immediately, two drops of sulphuric acid were added to stop further fermentation. The samples were then kept at 20 °C until they were further processed for VFA and ammonia determination. The second portion of the rumen fluid was immediately kept in ice and stored at − 20 °C until further analyses of microbial population study using a qPCR procedure.

For Experiment II, all animals were placed in an individual metabolic crate throughout the 19 days of the experiment (14 days of adjustment period to experimental diets and 5 days of sampling). The goats were divided into four groups of four goats and fed with the respective diets. They had free access to clean water. The respective diets were offered ad libitum to the goats at 09:00 daily until day 14. The feed intake of each animal on days 11–14 was recorded. On days 15–19, the animals were fed with 90% of the recorded intake. Fecal samples were collected daily from day 15 until day 19, and approximately 10% of the total collections were kept frozen at − 20 °C until further chemical analyses of nutrients. At the end of this study, all goats were fasted for 12 h with free access to drinking water, transported to the abattoir, allowed to rest, and then weighed before slaughter. The goats were slaughtered in accordance with the procedures outlined in MS1500:2009 (Department of Standards Malaysia, 2009) which allows animal to be slaughtered, without being stunned, with a razor sharp knife. In this study the slaughter was performed by a certified and highly experienced technician with a sharp knife. The goats were to be used for another study to determine the effects of different oils on carcass and meat quality which involves a food tasting study (not reported in the current study) [50].

### Chemical analyses of feed and fecal samples

Feed and fecal samples were analyzed for DM, OM, CP, and EE using the procedure by [48], whereas NDF and ADF were determined using the procedure by [49].

### Rumen pH and volatile fatty acid determination

The pH of the rumen content was measured immediately after the collection of the rumen fluid using a portable pH meter (Eco Testr pH 1, Eutech Instruments). For VFA determination, rumen filtrated samples were thawed at 4 °C prior to analysis following the procedure described by [51] with some modifications. One ml 3:1 *v*/v solution of 24% metaphosphoric acid and 5% formic acid was added into 5 ml of the rumen filtrate. The mixture was left to stand for 30 min before being centrifuged at 12,000 x g for 20 min. Then 0.5 ml of supernatant was collected and kept in 2-ml vials, and 0.5 ml internal standard (4-methyl-n-valeric acid) was pipetted into the vials. The samples were analyzed using gas chromatography, equipped with Flame Ionization Detector (FID) and capillary column (DB-FFAP, 122–3232).

### Ammonia determination

Rumen filtrated samples were centrifuged at 12,000 x g for 20 min. 5 ml of supernatant was collected and kept for further determination of the ammonia content using a protocol described by [52]. A standard solution was prepared using 1.908 g of ammonium chloride dissolved in 500 ml distilled water to give 1000 mg/l ammonia- nitrogen (ammonia-N). A standard 0.2, 0.5, 1.0, and 2.0 ppm solution were prepared by dissolving 0.02, 0.05, 0.10, and 0.20 ml of the stock solution with 100 ml distilled water, respectively. 5 ml of water (blank) or standard was added in an Erlenmeyer flask, and 0.2 ml of the phenol solution was added and swirled. In sequence, 0.2 ml of nitroprusside and 0.5 ml of oxidizing solution were added. The flask was then swirled, stopped and allowed to stand for 1 h at room temperature. The absorbance was then determined at 640 nm. Regression equation was determined from blank and standard samples before ammonia-N was estimated in the samples.

### DNA extraction and quantification using qPCR

Total genomic DNA from rumen content samples on day 0, day 12, and day 30 was extracted using QIAamp DNA Stool Mini Kit (Qiagen Inc., Valencia, CA, USA). The guideline on the protocol was provided by the manufacturer. The extracted DNA was stored at − 20 °C until subsequent procedures. Real-time PCR was used to determine the population of total bacteria, *F. succinogens*, *R. albus*, *R. flavefaciens*, methanogens, and protozoa. Species-specific PCR primers used to amplify partial 16S rDNA regions were chosen from literatures as presented in Table 5. Real-time PCR amplification and detection were performed using CFX 96 system (Bio-Rad, Hercules, CA, USA). The amplification reaction was conducted in a final volume of 25 μl containing 12.5 μl Maxima SYBR Green qPCR Master Mix, 1 μl species-specific PCR forward primer, 1 μl species-specific PCR reverse primer, 8.5 μl RNAse-free distilled water, and 2 μl of DNA elution. The PCR conditions of all species were as follows: an initial denaturation 95 °C for 15 s, followed by 39 cycles of denaturing of 15 s at 95 °C, 30 s at annealing temperature, and 20 s at 72 °C for an extension. The standards used in this study were prepared according to the protocol demonstrated by [53]. Briefly, DNA was extracted from a pure culture of microorganisms of interest to produce a high concentration of the target DNA using normal PCR. Later, the products of the PCR were purified using MEGAquick-spin™ (Intron Biotechnology, Inc.) The concentrations of the products were then measured using a Nanodrop ND-1000 spectrophotometer. An online formula [54] was used to calculate the number of copies of a template DNA per ml of elution buffer. Finally, standard curves were constructed using a serial dilution of plasmid DNA of each microbial group.

**Table 5:** The PCR primer used for quantification of rumen microorganism

Microbes	Primer		Amplicon (base pairs)	Ref
	Forward	Reverse		
General bacteria	5’-CGGCAACGAGCGCAACCC-3′	5’-CCATTGTAGCACGTGTGTAGCC-3′	130	[56, 57]
*Fibrobacter succinogenes*	5’-GTTCGGAATTACTGGGCGTAAA-3′	5’-CGCCTGCCCCTGAACTATC-3′	121	[56, 57]
*Ruminococcus albus*	5’-CCC TAA AAG CAG TCT TAG TTC G-3’	5’-CCT CCT TGC GGT TAG AAC A-3’	175	[58]
*Ruminococcus flavefaciens*	5’-CGAACGGAGATAATTTGAGTTTACTTAGG-3′	5’-CGGTCTCTGTATGTTATGAGGTATTACC-3′	132	[56, 57]
Methanogenic archea	5’-TTCGGTGGATCDCARAGRGC-3′	5’-GBARGTCGWAWCCGTAGAATCC-3′	140	[19]
Protozoa	5’-GCTTTCGWTGGTAGTGTATT-3′	5’-CTTGCCCTCYAATCGTWCT-3′	223	[19]

*Ref* References

### Determination of apparent digestibility

Apparent digestibility of each nutrient was calculated by measuring the feed intake and feces excreted. The feed and fecal samples were analyzed for nutrient of interest using similar procedures in the previous section of chemical analysis of feed and fecal samples. The differences between the amounts of nutrient consumed and excreted in the fecal samples are the amount of nutrient digested and absorbed:

$$ \mathrm{Apparent}\ \mathrm{digestibility}\ \left(\%\right)=\frac{amount\ of\ nutrient\ consumed- amount\ of\ nutrient\ excreted\ in\ faeces}{amount\ of\ nutrient\ consumed}\times 100 $$

### Statistical analysis

The sample size calculation for this study was established using the Resource Equation Approach.

The data for rumen fermentation and rumen microbial population were statistically analyzed using repeated measures of general linear model (GLM) procedure of [19]. It was used to analyze the parameters as affected by dietary treatments, days of sampling, and treatment × day of sampling interaction in the model. The Duncan multiple range test was used to further compare means at *P* < 0.05. The parameters for digestibility were analyzed using a one-way analysis of variance (ANOVA) using the GLM procedure of [55]. Mean differences were determined using the Duncan multiple range test at *P* < 0.05.

## Abbreviations

CNT: Control
OL: Olive oil
PO: Palm oil
SF: Sunflower oil
VFA: Volatile fatty acids
